# Impact of a Basic and Advanced Point-of-Care Ultrasound Training Program on Emergency Medicine Faculty Usage

**DOI:** 10.7759/cureus.112584

**Published:** 2026-07-13

**Authors:** Sarah K Kennedy, Frances M Russell, Loren Rood, Benjamin K Nti, Audrey Herbert, Matt Rutz, Daniel Brenner, Matthew Hays, Robinson M Ferre

**Affiliations:** 1 Emergency Medicine, Indiana University School of Medicine, Indianapolis, USA; 2 Biostatistics, Indiana University School of Medicine, Indianapolis, USA

**Keywords:** continuing medical education (cme), emergency medicine faculty, faculty development programs, point-of-care ultrasound (pocus), ultrasound utilization

## Abstract

Objective: Point-of-care ultrasound (POCUS) is an important diagnostic and procedural tool in emergency medicine. Although guidelines exist for POCUS training and credentialing, little data exist describing clinical utilization by practicing emergency physicians (EPs) after completion of residency or faculty training programs. The objective of this study was to evaluate the impact of basic and advanced POCUS training programs on subsequent clinical POCUS utilization by EPs.

Methods: This prospective observational cohort study included EPs who completed a basic and/or advanced POCUS training program developed internally according to established guidelines. Participants were followed for one year after training completion to assess clinical POCUS utilization, defined as the number of billed POCUS examinations. Associations between billed scan volume, perceived barriers, confidence, knowledge assessment scores, and objective structured clinical examination (OSCE) performance were evaluated using chi-square, Fisher's exact, and Kruskal-Wallis tests.

Results: A total of 138 EPs participated in the training program; 63 (46%) completed the basic program, and 75 (54%) completed the advanced program. Thirty-six (26%) faculty completed both. During the year following training, EPs in the basic cohort billed 814 POCUS examinations (median: three scans per physician, range: 0-185), while EPs in the advanced cohort billed 1,550 examinations (median: 10 scans per physician, range: 0-284). Confidence and knowledge improved following both training programs. However, billed POCUS utilization remained low. In the advanced cohort, greater comfort with ultrasound machine use was associated with increased clinical utilization.

Conclusions: Most EPs utilized POCUS for patient care after completing a training program. Despite improved confidence and knowledge, overall clinical use of POCUS was lower than expected. Competency-based POCUS training alone may be insufficient to drive sustained clinical adoption among practicing EPs. Further studies are needed to understand factors associated with increased physician POCUS integration.

## Introduction

Point-of-care ultrasound (POCUS) has become an integral component of everyday practice for many emergency physicians (EPs). The benefits of POCUS for patients and healthcare systems have been demonstrated through improved patient outcomes, decreased length of stay in the emergency department (ED), and reduced healthcare costs [1-5]. In 2001, the American College of Emergency Physicians published guidelines for POCUS use and credentialing in the emergency department [6]. Ten years later, the Residency Review Committee of the Accreditation Council for Graduate Medical Education adopted POCUS as a milestone in emergency medicine residency training [7,8]. However, many currently practicing EPs completed residency training before the widespread integration of POCUS education and therefore received limited or no formal ultrasound training [9].

While the majority of educational POCUS literature has focused on undergraduate and graduate medical education training [7,10,11], limited data exist studying faculty POCUS training pathways and resultant clinical utilization of POCUS among EPs [12-17]. This study aimed to assess the impact of basic and advanced training programs designed for practicing EPs on clinical utilization of POCUS. Additionally, we assessed factors associated with post-training utilization.

## Materials and methods

This was a prospective observational cohort study of EPs practicing across 10 different hospitals within a single healthcare system. Three sites were large urban academic hospitals, including one pediatric hospital, while the seven remaining were community hospitals. Details regarding the practice setting and the basic POCUS curriculum methods were previously published [11]. Data for this study were collected from 2016 to 2022. This study was deemed exempt by the institutional review board.

Basic POCUS training program

The basic POCUS training program included practicing EPs who did not meet the training program requirements during residency or clinical practice, as outlined by the American College of Emergency Physicians Ultrasound Guidelines [6]. This was defined as completion of 25 quality-reviewed exams in each of the core four POCUS modalities and a minimum of 150 total quality-reviewed studies. Participation in the program was voluntary. We excluded EPs with POCUS fellowship training or those who completed POCUS training in residency and met the basic training program requirements described above.

The basic POCUS training program began in 2016 and was subsequently offered several times annually over the course of three additional years between 2018 and 2020. While this was first implemented for practicing EPs at the three academic teaching hospitals, the curriculum was later expanded in a staggered fashion to include EPs practicing at seven community hospitals within the healthcare system. Completion of the basic POCUS training program required 16 hours of POCUS-related continuing medical education, including an in-person, hands-on workshop, ≥ 25 quality-reviewed POCUS examinations in each of four core modalities, and passing a knowledge test. For EPs working in EDs that saw patients of all ages, the basic POCUS training program included four modalities: abdominal aorta, first-trimester obstetric, extended focused assessment with sonography in trauma (eFAST), and cardiac ultrasounds. For EPs who worked in a pediatric ED, the POCUS modalities included cardiac, lung, soft tissue, and eFAST exams. All faculty members were required to pass a knowledge assessment with a minimum score of 70% to complete the training program. Those who did not pass were allowed to remediate knowledge gaps in training and retake the exam, although only their initial score was used for data analysis.

Pre- and post-training assessments were administered to evaluate changes in POCUS confidence, perceived barriers to use, and knowledge. A pre- and post-survey was administered as part of each workshop, and a post-survey was given after completion of the basic or advanced training programs. These surveys were developed internally by the ultrasound division to collect demographic information, perceived barriers to POCUS use, and personal comfort with ultrasound performance and interpretation. If a faculty member responded “yes” to a barrier, it indicated that they perceived it as a barrier to using POCUS. Demographic information included years since residency training, their self-reported current ultrasound use, and the estimated number of previously performed ultrasounds. Participants were asked to identify perceived barriers to using ultrasound in the ED, including an “other” section with the option for respondents to provide a free-text response. Comfort with ultrasound use was assessed with Likert-scale questions evaluating confidence with machine use, image acquisition and interpretation, and clinical integration. While not validated, surveys were developed using publications available at the time of creation [18,19]. Beginning in 2019, an objective structured clinical examination (OSCE) was implemented following the initial hands-on workshop to evaluate faculty psychomotor and image acquisition skills. The OSCE included the modalities taught during the workshop and was administered by ultrasound faculty. Learners were required to demonstrate machine knowledge by selecting the correct probe and machine settings and entering patient information. They then needed to locate structures on standardized patients and verbalize anatomy. A grade was assigned on the OSCE form based on the learner's ability to perform the tasks, obtain quality images, and interpret those images correctly. No assistance was given during the exam, but feedback was provided afterwards if requested or if the learner was unable to complete a question. Completion of the basic POCUS training was incentivized by the Department Chair through a bonus structure. Curriculum details and supplemental materials were previously published [17].

Advanced POCUS training program

The advanced POCUS training program included EPs who had completed either the basic training program or the residency-based or practice-based training pathway, as outlined by the American College of Emergency Physicians Ultrasound Guidelines [6]. We excluded faculty with POCUS fellowship training. The advanced program required completing ≥ 200 quality-reviewed POCUS exams across all modalities and attendance at one advanced POCUS workshop. Three advanced workshops were offered multiple times annually from 2019 to 2022. Similar to the basic curriculum, the advanced workshops included didactics and hands-on skills stations, a pre- and post-knowledge assessment, a pre- and post-survey, and a post-workshop OSCE skills assessment. This curriculum has been previously described [17]. Workshops were taught by fellowship-trained POCUS faculty and lasted 6.5 hours. Advanced workshops covered the following modalities: soft tissue, ocular, vascular access, gallbladder, renal, thoracic, deep venous thrombosis (DVT), musculoskeletal, nerve blocks, advanced cardiac, and pediatric abdomen ultrasound applications. Faculty could attend more than one workshop. Only data from the initial workshop participation were included in the analysis. 

Clinical POCUS utilization

POCUS utilization by EPs was assessed one year after completion of either basic POCUS training or attendance at an advanced POCUS workshop and was determined by counting the number of billed POCUS scans completed by faculty during this time. In the subset of faculty who completed both the basic and advanced courses, we counted their scans after the basic course in 2016 and then again after their advanced course, which occurred over a year later. Billed scans were identified through a systematic review of the institutional POCUS workflow solution (QPath, Telexy). In the academic setting, billing for POCUS scans required a faculty member to have met ACEP ultrasound training guidelines [6] and either acquire images themselves or supervise trainee image acquisition. Supervision could be done in real time at the bedside or through image review. Faculty members then interpreted the images and completed the billing workflow. At our institution, departmental leadership recommends that all clinically indicated POCUS examinations be documented and submitted for billing through the institutional POCUS workflow. Examinations that were educational only or were not clinically indicated were not submitted for billing and were excluded from analysis.

Data analysis

Separate cross-sectional analyses were conducted for the basic and advanced cohorts. Basic and advanced workshops were not directly compared; all comparisons remained within the respective workshop at a significance level of alpha = 0.05. For subjects from each workshop, chi-square and Fisher's exact tests were conducted to determine a significant association between the number of scans billed and POCUS barriers, as well as comfort with POCUS. The Kruskal-Wallis test was used to assess distributions of the number of barriers relative to the number of scans performed clinically. Fisher's exact test was used to determine whether there was a significant association between the knowledge assessment score and OSCE assessment score and the number of scans billed. We did not perform a sample size calculation as this study was exploratory in nature and included all eligible participants available during the study period.

## Results

A total of 138 EPs participated in the training program; 63 (46%) completed the basic program, and 75 (54%) completed the advanced program. Thirty-six (26%) faculty completed both. The majority of EPs worked primarily in an academic setting; see Table 1 for demographics. The mean time since residency graduation for EPs was 19 years.

**Table 1:** Emergency medicine physician demographics. Data are presented as n and %. Missing data for one person in the basic workshop and one person in the advanced workshop. FTE = full-time equivalent

Training level	Basic, n=63*	Advanced, n=75*
Gender, male	41 (65%)	42 (56%)
Years post-residency training, mean (standard deviation)	19 (8.5)	14 (8.7)
Academic or community site		
Academic	42 (68%)	49 (66%)
Community	20 (32%)	18 (24%)
Split (academic and community)	0	7 (10%)
Adult or pediatric site		
Adult	53 (85%)	54 (73%)
Pediatric	6 (10%)	13 (17%)
Split (adult and pediatric)	3 (5%)	7 (10%)
Full-time equivalent (FTE)		
Full time	53 (85%)	66 (89%)
Part time (<0.75 FTE)	9 (15%)	8 (11%)
Number of ultrasounds previously performed		
0-25	18 (29%)	0
26-100	20 (32%)	0
>100	24 (39%)	74 (100%)

Basic POCUS training

For attendees of the basic workshops, 18 (29%) had performed fewer than 25 POCUS examinations, and 5 (8%) faculty had never performed POCUS prior to basic training. The most commonly reported pre-training barriers were unfamiliarity with the workflow for submitting and billing clinical POCUS exams and comfort level in personal skills. After training, there was a significant reduction in barriers related to ultrasound machine operation (p<0.001), image interpretation (p=0.021), and submission of images for quality assurance review (p<0.001). Confidence in obtaining, interpreting, and incorporating POCUS into clinical practice significantly improved after training completion (p<0.001) (see Table 2).

**Table 2:** Basic workshop barriers and confidence assessment. Data are presented as n and % for barriers and median and range for confidence. Pre- to post-confidence was analyzed using a paired t-test. Pre- to post-barriers were analyzed using McNemar's test for each barrier separately. P-values <0.05 were considered significant. US = ultrasound

Barriers, n (%)	Pre-workshop, n=63	Post-workshop, n=63	p-value
Don’t know how to use the machine	19 (30)	4 (6)	<0.001
Don’t know how to interpret images	21 (33)	11 (17)	0.021
Comfort level of personal skills	39 (62)	35 (56)	0.48
Don’t see the utility of using it	3 (5)	1 (1.6)	0.5
Don’t know how to use the workflow solution	35 (56)	7 (11)	<0.001
None	4 (6)	9 (14)	0.062
Confidence, median (range)	n=63	n=62	p-value
I feel confident using the US machine	3 (1-5)	4 (2-5)	<0.0001
I feel confident obtaining US images	3 (1-5)	4 (2-5)	<0.0001
I feel confident interpreting US images	3 (1-5)	4 (2-5)	<0.0001
I feel confident incorporating the US into clinical practice	3 (1-5)	4 (2-5)	<0.0001

Twenty-five out of 63 (40%) EPs completed both pre- and post-workshop POCUS knowledge assessments. Median scores improved from 67% pre-workshop to 79% post-workshop, although this did not reach statistical significance (p=0.087). Thirty-three out of 63 (52.4%) EPs completed a post-workshop OSCE, with an average score of 88.3% (range: 59-100%).

Longitudinal results for basic POCUS training

A total of 814 billed POCUS scans were completed by EPs within one year of completing basic POCUS training, with a median of three scans per physician (range: 0-185). Twenty (31%) of EPs did not complete any billed POCUS scans after training. No measured variables, including perceived barriers, confidence levels, knowledge, or OSCE scores, were significantly associated with performing billed POCUS exams after training completion (see Table 3).

**Table 3:** Association of factors and ultrasound utilization. Data are presented as p-values. Chi-square and Fisher's exact tests were conducted to determine a significant association between barriers and confidence and utilization. The Kruskal-Wallis test was used to assess the number of barriers and utilization. Fisher's exact test was used to determine whether there was a significant association between post-knowledge assessment scores and OSCE scores and utilization. P-values <0.05 were considered significant. US = ultrasound; OSCE = objective structured clinical examination

Training level	Basic (p-value)	Advanced (p-value)
Barriers		
Takes too much time	0.1711	0.5465
Don't know how to use the machine	1	0.4934
Don't know how to interpret images	0.263	0.6841
Comfort level of personal skills	0.2712	0.2997
I don't see the utility of using it	0.4286	0.2345
Lack of machine availability	1	0.7544
Too busy/time-consuming	0.2152	0.8118
Don't know how to submit on QPath	1	0.1857
None	0.6884	0.1378
Number of barriers	0.2753	0.5634
Confidence		
Using the US machine	0.6854	0.0413
Obtaining US images	0.6748	0.0891
Interpreting US images	0.5679	0.3749
Incorporating US into clinical practice	0.7055	0.0978
Post-Knowledge Score	0.0526	0.7454
OSCE Score	0.6351	0.1805

Advanced POCUS training results

For the advanced training cohort of EPs, we found no significant reduction in reported barriers to POCUS use post-workshop, although all barriers were relatively low to begin with, except comfort level in personal skills, which remained high before and after the workshop. All areas of confidence with POCUS improved (p<0.001) after the workshop (see Table 4).

**Table 4:** Advanced workshop barriers and confidence assessment (n=75). Data are presented as n and % for barriers and median and range for confidence. Pre- to post-confidence was analyzed using a paired t-test. Pre- to post-barriers were analyzed using McNemar's test for each barrier separately. P-values <0.05 were considered significant. US = ultrasound

Barriers, n (%)	Pre	Post	p-value
Don’t know how to use the machine	8 (11)	8 (11)	1
Don’t know how to interpret images	23 (31)	18 (24)	0.27
Comfort level of personal skills	43 (57)	40 (53)	0.55
Don’t see the utility of using it	4 (5)	2 (3)	0.5
Don’t know how to use the workflow solution	10 (13)	13 (17)	0.45
None	1 (1)	4 (5)	0.25
Confidence, median (range)			
I feel confident using the US machine	4 (2-5)	4 (3-5)	<0.0001
I feel confident obtaining US images	4 (2-5)	4 (2-5)	<0.0001
I feel confident interpreting US images	3 (1-5)	4 (2-5)	<0.0001
I feel confident incorporating US into clinical practice	3 (2-5)	4 (2-5)	<0.0001

Seventy-three out of 75 (97%) EPs completed a pre- and post-advanced workshop POCUS knowledge assessment. Knowledge scores significantly improved from pre- to post-workshop, 70% vs 87% (p<0.001). All EPs completed a post-workshop OSCE with an average score of 95.3% (range: 79-100%).

Advanced longitudinal results

A total of 1,550 billed POCUS scans were completed by EPs after advanced POCUS training with a median of 10 per physician (range: 0-284). Twelve (16%) EPs did not complete any billed scans after training. The only factor found to be a significant predictor of billed scans was comfort with ultrasound machine use (p=0.041), with higher comfort levels associated with increased post-training utilization (see Table 3). No other factors were significantly associated with billed scan volume.

## Discussion

POCUS is an essential tool for EPs and can assist with diagnosis, procedural guidance, and patient management in the ED. As many practicing EPs completed residency before the widespread incorporation of POCUS education, it is important to understand how the implementation of faculty training and guideline recommendations impacts knowledge, skill acquisition, and clinical utilization for these physicians [6]. In this study on 138 EPs from a single healthcare system, implementation of basic and advanced POCUS training programs was associated with improvements in physician knowledge and confidence, as well as reductions in several self-reported barriers to use for the basic cohort of EPs. EPs demonstrated a high ability to obtain images and identify pertinent anatomy for both the basic (88%) and advanced cohorts (95%). Importantly, the majority of faculty utilized POCUS post-training in the clinical setting.

The primary finding of this study was POCUS utilization in the clinical setting, measured by the number of billed POCUS examinations after the curriculum ended. The basic cohort billed 814 exams within a year after curriculum completion, and the advanced cohort billed 1,550 exams. However, utilization was highly variable between physicians. Median billed scan volume remained low at three scans per physician in the basic cohort and 10 scans per physician in the advanced cohort, and 32 physicians did not bill a single POCUS examination after training completion. It is noteworthy that one faculty completed 185 examinations alone in the basic cohort and another billed 284 examinations alone in the advanced cohort. These findings suggest that improvements in confidence and competency alone may be insufficient to drive sustained clinical adoption of POCUS among practicing EPs.

Few prior studies have evaluated EP utilization of POCUS after the implementation of a training program. In 2020, Smalley et al. retrospectively evaluated POCUS utilization among community EPs [13]. In this study, over a two-year period, 5,099 scans were performed by 170 faculty across 11 emergency departments. The majority of billed POCUS exams were completed by newly graduated EPs presumably trained with POCUS during residency, although not specified. The volume of scans decreased in EPs who were further from residency training. In both the Smalley et al. study [13] and our cohort, individual physician scan volumes were lower than expected relative to the number of participants and patient volumes.

In 2022, Nti et al. studied the impact of a POCUS curriculum on the management of pediatric soft tissue infections in the ED [15]. In this before-and-after study on 119 patients, POCUS utilization significantly increased post-POCUS implementation, leading to reduced ED length of stay and decreased costs. This aligns with our study and others showing increased POCUS utilization after an intervention, although the number of faculty utilizing POCUS after training remains small [20,21].

An important contribution of this study was the evaluation of factors associated with post-training utilization. Among the variables examined, only physician comfort with ultrasound machine operation was significantly associated with increased billed scan volume, and this association was observed only in the advanced cohort. Clinical utilization was not predicted by self-reported barriers, knowledge assessment scores, or OSCE performance. Interestingly, even physicians reporting no perceived barriers to POCUS use were not more likely to utilize POCUS clinically. These findings suggest that factors beyond technical competency may play a substantial role in determining whether physicians incorporate POCUS into routine practice.

Although billed examinations were selected as an objective measure of utilization, these values may underestimate true clinical POCUS use. Several explanations may account for the relatively low utilization observed. Billing required the system to be functioning properly and to have successful completion of the workflow, including saving images, documentation, and submission on the platform. Despite the curriculum including education on the workflow process, physicians may not have completed these steps in clinical practice. Operational barriers, including workflow interruptions, technical issues, time constraints on busy shifts, and discomfort with exams or billing, may have all contributed to reduced use and documentation. Physicians may lack confidence in using POCUS findings to guide clinical decision-making despite improved technical skills. Additionally, some physicians may not perceive sufficient clinical utility to justify routine use. Many of these barriers have been previously described in the literature [19]. It is likely that the true clinical utilization was higher than the number of billed exams captured in this study. Collectively, these findings suggest that successful implementation of POCUS among practicing EPs likely requires more than competency-based education alone and may depend on workflow integration, institutional culture, ongoing mentorship, and reinforcement of clinical application. They highlight the need for further investigation into factors associated with increased utilization, in order to better design a POCUS curriculum and allocate resources.

Limitations

Several limitations should be considered that may affect the generalizability of this study. This was a single healthcare system. However, the scale of this study included various subgroups, including community, pediatric, and academic departments, which could improve applicability to a broader range of EP settings. It is important to note that a financial incentive was provided for completion of the basic curriculum. This was not tied to a requirement to submit billed ultrasound exams, but this incentive may have influenced participation and the feasibility of curriculum implementation, which may not accurately reflect institutions without similar resources or support. However, this is consistent with prior studies that support incentivizing faculty training [16,22]. A major limitation of this study is the lack of baseline POCUS utilization data. Although baseline utilization among faculty was known to be low, as evident in Table 1, pre- and post-implementation changes in clinical POCUS use were not systematically tracked because this project was initially designed as a quality improvement initiative to evaluate the educational components of the curriculum rather than changes in clinical practice. Future prospective studies should evaluate the impact of faculty development curricula on POCUS utilization before and after implementation. Participation in post-training assessments was incomplete, including surveys, knowledge assessments, and OSCE evaluations, which introduces the possibility of selection bias. Faculty who completed the evaluations may have been more engaged and motivated to learn and use POCUS, potentially biasing results towards more favorable outcomes. While the educational curriculum remained consistent throughout the study period, an OSCE was implemented in 2019 to evaluate learner competency rather than to alter the educational intervention itself. Importantly, we found no evidence that the introduction of the OSCE was associated with differences in subsequent POCUS utilization, suggesting that this change in assessment methodology was unlikely to have meaningfully influenced the primary outcome. While feedback was not provided to participants during the evaluation, hands-on assessments may have influenced subsequent utilization or performance by participants. Finally, billed POCUS examinations were used as a surrogate measure for clinical POCUS utilization. Accurate billing volumes were dependent upon completion of the institutional POCUS workflow, including image saving, documentation, and study submission on the platform. Consequently, the number of billed examinations likely underestimates the true number of clinically performed POCUS examinations.

## Conclusions

In this mixed cohort of academic, community, and pediatric EPs from a single healthcare system, implementation of basic and advanced POCUS training programs was associated with improved physician knowledge and confidence, as well as reduced perceived barriers to use. Despite these improvements, overall clinical POCUS utilization, as measured by billed examinations, remained modest. These data would suggest that comfort with ultrasound machine operation was the only factor associated with increased clinical utilization. Although competency-based training improved physician confidence and knowledge, additional organizational, environmental, and behavioral factors likely influence long-term POCUS adoption. Further research is needed to identify educational, operational, and systems-based factors that support long-term integration of POCUS into clinical practice.
